# Selective Deactivation of Serum IgG: A General Strategy for the Enhancement of Monoclonal Antibody Receptor Interactions

**DOI:** 10.1016/j.jmb.2012.04.002

**Published:** 2012-06-29

**Authors:** Kavitha Baruah, Thomas A. Bowden, Benjamin A. Krishna, Raymond A. Dwek, Max Crispin, Christopher N. Scanlan

**Affiliations:** 1 Oxford Glycobiology Institute, Department of Biochemistry, University of Oxford, South Parks Road, Oxford OX1 3QU, UK; 2 Division of Structural Biology, Wellcome Trust Centre for Human Genetics, University of Oxford, Roosevelt Drive, Oxford OX3 7BN, UK

**Keywords:** ADCC, antibody-dependent cellular cytotoxicity, FcγR, Fcγ receptor, mAb, monoclonal antibody, PDB, Protein Data Bank, sAb, serum antibody, antibody, endoglycosidase, Fc receptor, ADCC, glycosylation

## Abstract

Serum IgG is a potent inhibitor of monoclonal antibody (mAb) binding to the cell-surface Fcγ receptors (FcγRs), which mediate cytotoxic and phagocytic effector functions. Here, we show that this competition can be eliminated, selectively, by the introduction to serum of (i) an enzyme that displaces Fc from FcγRs and (ii) a modification present in the therapeutic mAb that renders it resistant to that enzyme. Specifically, we show that (i) EndoS (endoglycosidase S) cleaves only complex-type glycans of the type found on IgG but (ii) is inactive against an engineered IgG Fc with oligomannose-type glycans. EndoS thus reduces FcγR binding of serum IgG, but not that of engineered mAb. Introduction of both the engineered mAb and endoglycosidase in serum leads to a dramatic increase in FcγR binding compared to the introduction of mAb in serum alone. Antibody receptor refocusing is a general technique for boosting the effector signal of therapeutic antibodies.

## Graphical Abstract

**Figure:**
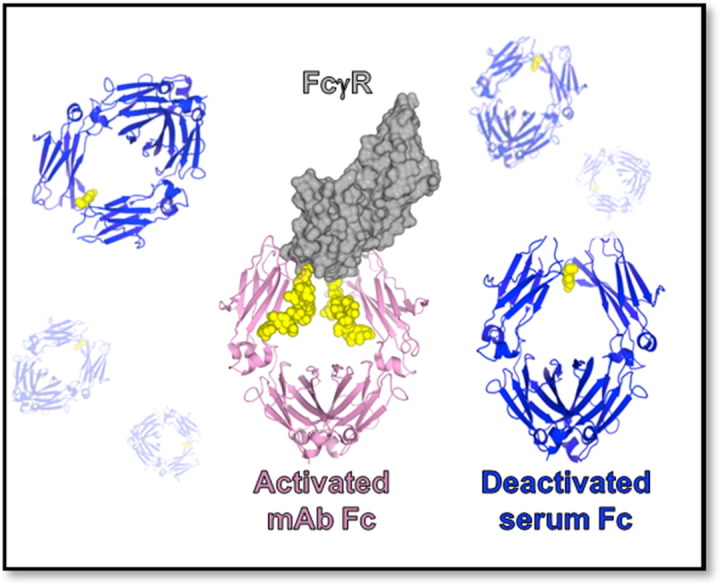

**Highlights:**

► Therapeutic antibodies compete against serum antibodies for cellular Fc receptors. ► We report antibody glycoforms resistant to deactivating endoglycosidases. ► Selective elimination of serum antibodies enhances mAb effector functions.

The ability of an antibody to recruit the killing machinery of the cellular immune system is dependent on the interaction between the antibody Fc domain and Fc receptors found on cells such as macrophages and natural killer cells.^1^ This antibody-dependent cellular cytotoxicity (ADCC) is required for the efficient elimination of cancerous or infected cells. The binding of Fc to Fcγ receptors (FcγR) bridge the adaptive and innate immune systems and is a primary focus of therapeutic monoclonal antibody (mAb) research.^2,3^

The affinity for each FcγR is different for each of the naturally occurring isotopes of IgG (IgG1–4).^4^ Similarly, artificial point mutations in the receptor-binding interface (notably, the lower hinge and upper loops of the Cγ2 regions) exhibit a wide range of modified affinities for both activating and inhibitory receptors.^5^ The strength of Fc:FcγR interaction is also highly dependent on the presence and composition of the dynamic N-linked glycans attached to Asn297 of both of the Cγ2 domains of the IgG Fc homodimer.^6–9^ For example, de-fucosylation increases ADCC, principally by reducing unfavorable steric interactions between the Fc glycan and the glycan at Asn164 of the activating FcγRIIIa receptor found on natural killer cells.^9–11^ One well-explored approach to enhance effect or function is therefore to increase the intrinsic affinity for FcγR by engineering the Fc structure of a given mAb.^12^ However, an additional factor also impacts Fc:FcγR interactions: the presence of competing serum IgG.

Serum antibody (sAb) (50–100 μM) is present in significant excess of the dissociation constant (*K*_d_) for IgG Fc:FcγR interactions (0.1–10 μM).^13,4^ The majority of cellular FcγRs are therefore bound to IgG Fc under physiological conditions. For example, FcγRIIIa (Val158 variant) exhibits a *K*_d_ of around 0.1 μM for IgG1 Fc.^14^ Therefore, regardless of the affinity of an Fc for an FcγR, the limited availability of unbound FcγR imposes an external constraint on antibody effect or potency. While Fc engineering can help overcome this effect for antigens expressed at high levels on the cell surface,^15^ low-affinity or low-copy epitopes on infected or cancerous cells are efficiently protected from ADCC by serum immunoglobulins.^13,15,16^ This leads to dosages of clinical therapeutic antibody several orders of magnitude greater than would be predicted by serum-free assays.^16^

We designed an assay to replicate how mAb binding to the FcγRIIIa receptor is affected by human serum. In order to allow selective detection of the engineered antibody Fc within an excess of serum Fc domains, we constructed a chimeric mAb containing the full human IgG1 Fc and a murine Fab region (Supplementary Methods) allowing detection with an anti-murine Fab secondary antibody (Fig. 1a). Consistent with previous reports,^13,15^ even at extensive serum dilutions, mAb binding was significantly reduced, and at serum concentrations approaching physiological levels, mAb binding was barely detectable. Therefore, the elimination of competing sAb represents a route to the enhancement of mAb:FcγR interactions.

One solution in circumventing the inhibitory effect of sAb is to selectively eliminate serum IgG binding to FcγRs while leaving therapeutic mAb function unperturbed. A number of bacterial enzymes are able to interrupt Fc:FcγR interactions.^23^ Notably, secreted endoglycosidases are able to cleave the core GlcNAcβ1 → 4GlcNAc linkage of the Fc glycan, release IgG from cellular FcγRs and abrogate Fc-mediated effector function.^24–27^ To elucidate the molecular basis of IgG deactivation by endoglycosidases, we deglycosylated IgG1 Fc to a single GlcNAc moeity and determined its structure by X-ray crystallographic analysis to a resolution of 2.5 Å (Fig. 1b and Table 1 and Supplementary Methods and Fig. S1). Comparison of this Fc^GlcNAc^ with known glycosylated IgG Fc structures reveals that endoglycosidase cleavage induces an inward movement of the Cγ2 domains coupled with a rotation around the central axis (Fig. 1b). This structural transformation displaces equivalent C_α_ atoms by up to 8 Å. By analogy with the conformation observed in aglycosylated murine IgG Fc^29^ (Supplementary Fig. 1), the fully closed quaternary structure observed here is incompatible with known Fc:FcγR interactions.^30^ This structure is consistent with solution-phase NMR data that reveal significant changes in chemical shifts from the Cγ2 domain residues following endoglycosidase cleavage.^31^ Similarly, displacement of the C'E loop in our Fc^GlcNAc^ structure provides a plausible explanation for the altered hydrogen/deuterium exchange kinetics reported in this region following deglycosylation.^32^ We also note that mutations able to restore functionality to aglycosylated antibodies include residues in the C'E loop.^33^

The change in conformation seen in our Fc^GlcNAc^ structure also offers a structural basis for natural immune evasion by a common human pathogen. EndoS (endoglycosidase S), from *Streptomyces pyogenes*, deglycosylates human IgG and decreases FcγR binding of antibacterial antibodies,^24–27^ an observation confirmed here for FcγRIIIa (Fig. 1c). The activity of this enzyme has also been employed for therapeutic applications: EndoS is under preclinical development as an immunosuppressive agent to *diminish* antibody-mediated pathology via elimination of Fc:FcγR interactions of autoimmune antibodies.^34^ However, we hypothesized that EndoS could also be used to *enhance* binding of mAbs to FcγR *provided deactivation was focused to bulk serum IgG and not to recombinant mAb*. This would require an engineered antibody that maintains productive FcγR binding with a carbohydrate component unaffected by EndoS.

The carbohydrate specificity of EndoS is not known.^35^ However, consideration of its evolved function suggested that commonly occurring IgG Fc glycoforms would be efficiently hydrolyzed.^26^ By contrast, it seemed less likely that EndoS would have acquired activity against glycans not normally found on human IgG. For example, oligomannose-type structures are devoid of the terminal carbohydrate motifs typically present on antibody glycans (principally, NeuNAcα2 → 6Gal, Galβ1 → 4GlcNAc and GlcNAcβ1 → 2Man). Fortuitously, however, oligomannose Fc glycoforms exhibit high-affinity binding to all human FcγRs^14,36,37^ and serum clearance equivalent or slightly reduced^38^ compared to complex-type glycoforms.

The ability of EndoS to hydrolyze either naturally glycosylated IgG1 Fc or an engineered Fc bearing oligomannose-type glycans was therefore determined. An advantage of the particular Man_5_GlcNAc_2_ Fc glycoforms is that chemically homogenous glycoproteins can be readily manufactured at high yields through manipulation of the mammalian glycan biosynthetic pathway.^37,39,40^ Mass spectrometric analysis of complex-type *N*-glycans, released from IgG1 Fc and subsequently exposed to EndoS, showed complete cleavage of the core GlcNAcβ1 → 4GlcNAc linkage (Fig. 2a and b). In contrast, the oligomannose-specific EndoH (endoglycosidase H) showed no detectable hydrolysis (Fig. 2c). A reciprocal pattern of specificities was observed for an engineered oligomannose Fc glycoform that displayed complete resistance to EndoS (Fig. 2d–f).

The enzymatic resistance of oligomannose-type mAb provides a route to selective elimination of natural antibody glycoforms from cellular FcγRs. We therefore repeated our FcγR binding assay using an oligomannose mAb glycoform, in the presence of serum and EndoS, EndoH, both EndoS and EndoH or no enzyme (Fig. 3a). Consistent with the data from Fig. 1, the enzyme-free serum efficiently blocked the binding of the oligomannose-type mAb to FcγRIIIa. However, the addition of EndoS led to a dramatic increase in apparent affinity of oligomannose mAb for FcγRIIIa with 50% receptor saturation achieved at approximately 0.05 μM mAb, a level approaching that of mAb:FcγR determined for IgG in the complete absence of serum and consistent with the reported value (0.08 μM) of this interaction.^4^ This enhancement was a direct consequence of the differential glycosylation of the engineered mAb and natural sAb. This glycoform dependence was confirmed by the addition of EndoH, which led to loss of detectable FcγRIIIa binding regardless of whether or not the competing sera had also been treated with EndoS. These data indicate that it is possible to selectively target Fc receptors to IgGs with specific glycoforms despite a large excess of competing serum Fc (Fig. 3b). The well-documented biological and pharmacological properties of both the oligomannose glycoforms and the endoglycosidase enzymes point toward the *in vivo* development of this approach for almost any Fc:FcγR-dependent process. Similarly, any EndoS-resistant antibodies including aglycosylated mAbs engineered to exhibit functional FcγR interactions^33^ could be employed. An obvious additional application of mAbs, resistant to IgG deactivating enzymes, would be in the treatment of infections by bacteria, such as *S. pyogenes*, which secrete these immune evasion factors.

While mammalian glycosylation is heterogeneous, manipulation of cellular glycan biosynthesis can yield chemically homogenous, precisely defined protein glycoforms. Similarly, despite cleaving the same core GlcNAcβ1 → 4GlcNAc linkage conserved in all N-linked glycans, endoglycosidases have evolved a remarkable selectivity for terminal carbohydrate motifs. In combination, these tools allow for precise and independent control of natural and engineered glycoproteins, even in the complex biochemical environment of human serum. Our strategy, which we term *receptor refocusing*, provides a general approach for boosting the immunological signal provided by mAbs: by redirecting the cellular immune system to a single antibody glycoform, which is in turn directed to a single target antigen.

## Accession codes

Coordinates and structure factors of Fc^GlcNAc^ have been deposited in the Protein Data Bank (PDB accession number 4ACP).

## Author Contributions

K.B., M.C., R.A.D. and C.N.S. designed the experiments. K.B. and T.A.B. designed and performed the crystallographic experiments. K.B., B.A.K. and C.N.S. performed glycan analysis and ELISA experiments. K.B., T.A.B., M.C. and C.N.S. wrote the manuscript.

## Conflict of Interest

The authors declare no competing financial interests.

## Figures and Tables

**Fig. 1:**
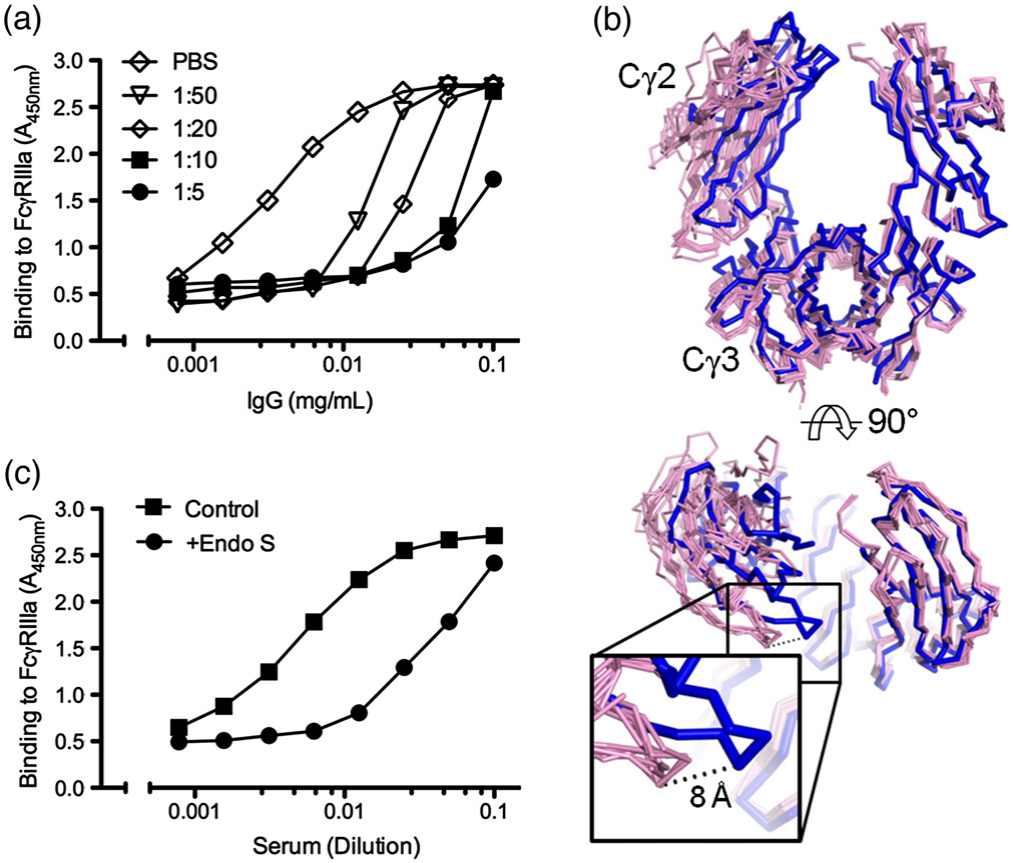
Endoglycosidase-mediated deactivation of serum IgG. (a) Binding of human IgG1 Fc to immobilized FcγRIIIa was determined, by ELISA, in the presence of PBS (*p*hosphate-*b*uffered *s*aline) or increasing concentrations of human serum and detected using a secondary antibody specific for the monoclonal Fab domain (Supplementary Methods). (b) The crystal structure of Fc^GlcNAc^ (blue ribbon) overlaid with structures of glycosylated human IgG Fcs using SHP^17^ by superposition of C_α_ residues from one protomer while leaving the second protomer free (pink ribbons; PDB IDs 1FC1, 1H3T, 1H3U, 1H3V, 1H3W, 1H3X, 1H3Y, 2DTQ, 2DTS, 3DNK, 3D03 and 3HKF). Broken lines are drawn between equivalent C_α_ atoms (Tyr296) in Fc^GlcNAc^ and naturally glycosylated structures and indicate a displacement of approximately 8 Å. For crystallographic analysis, Fc^GlcNAc^ (Supplementary Methods) was concentrated to 7.0 mg/mL and was crystallized after 42 days with the use of the sitting-drop vapor diffusion method^18^ using 100 nL protein plus 100 nL precipitant equilibrated against 95 μL reservoirs. Crystals grew at room temperature in a precipitant containing 25% (w/v) polyethylene glycol 1500 and 0.100 M SPG System buffer (pH 4). Crystals were flash frozen by immersion in a cryoprotectant containing the mother liquor diluted in 25% (v/v) glycerol and then rapidly transferred to a gaseous nitrogen stream. X-ray diffraction data were recorded at beamline I03 at Diamond Light Source, Oxfordshire, England. Data were processed and scaled using DENZO and SCALEPACK,^19^ and the structure was solved using Phaser^20^ with native Fc (PDB accession number 3AVE) as a search model. Model building was performed with Coot^21^ and iteratively refined using restrained refinement with TLS in the CCP4 supported program REFMAC5.^22^ (c) Binding of EndoS-treated or mock-treated human sera to immobilized FcγRIIIa determined using an anti-human IgG secondary antibody (Supplementary Methods).

**Fig. 2:**
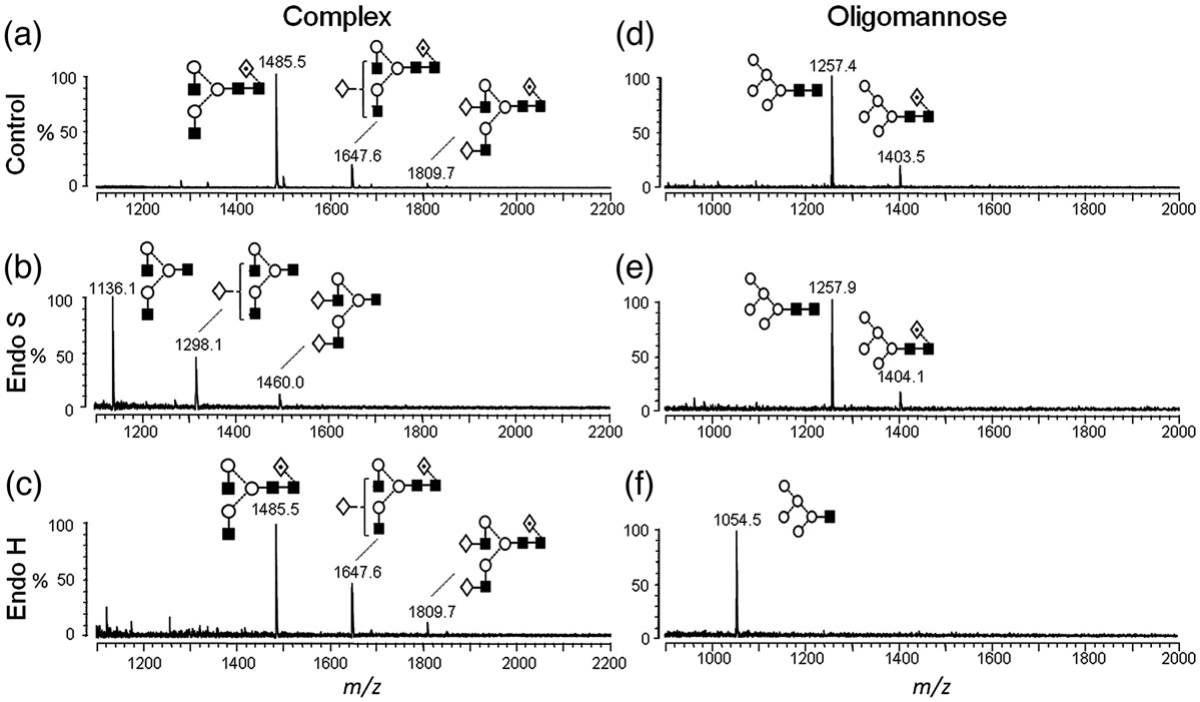
Resistance of oligomannose containing Fc glycoforms to EndoS-mediated hydrolysis. Matrix-assisted laser desorption/ionization mass spectrometry spectra of PNGase-F released *N*-glycans from IgG1 Fc expressed in GnT-I-deficient HEK (*h*uman *e*mbryo *k*idney) 293S cells (Supplementary Methods). These expression systems yielded, respectively, Fc with complex-type (a–c) or oligomannose-type (d–f) glycans, which were exposed to no enzyme (a and d), EndoS (b and e) or EndoH (c and f). The spectra of the oligomannose glycans reveal the presence of GnT-I-independent fucosylation.^41^ The cleavage of the core GlcNAcβ1 → 4GlcNAc bond by endoglycosidases results in the removal of a single GlcNAc (predicted Δ*m*/*z* = 203.1) or Fucα1 → 6GlcNAc (predicted Δ*m*/*z* = 349.1). Symbolic representation of glycan structures follows that of Harvey *et al*.:^42^ ◊,   Gal; ■,   GlcNAc; ○,   Man; ,   Fuc. The linkage position is shown by the angle of the lines linking the sugar residues (vertical line,   2-link; forward slash,   3-link; horizontal line,   4-link; back slash,   6-link). Anomericity is indicated by continuous lines for β-bonds and by broken lines for α-bonds.

**Fig. 3:**
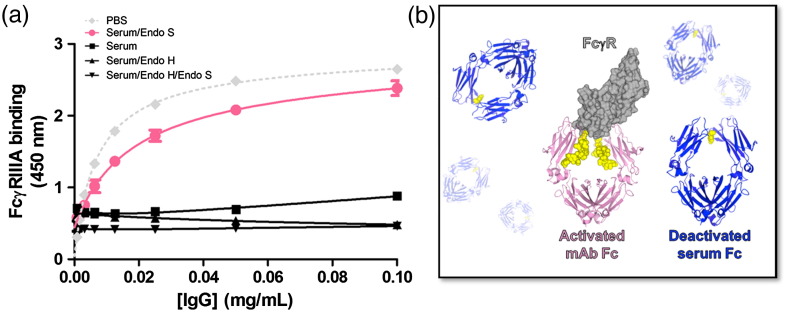
EndoS-mediated deactivation of serum leads to enhancement of mAb binding to FcγRIIIa. (a) ELISA showing the interaction between monoclonal IgG1 containing oligomannose (Man_5_GlcNAc_2_) glycans and immobilized FcγRIIIa in the presence of PBS, serum, serum and EndoS, serum and EndoH or serum and EndoS and EndoH (Supplementary Methods). Binding was detected using a secondary antibody specific for the monoclonal Fab domain as in Fig. 1a. Data points represent the calculated mean of three independent measurements from a total of four experiments. (b) Schematic illustration of the differential binding of FcγRIIIa to oligomannose and natural Fc glycoforms in the presence of EndoS. Deactivated Fc^GlcNAc^ is shown in blue, and activated Fc in complex with FcγRIIIa (gray surface; PDB ID 1T83) is shown in pink. Glycans are shown as yellow spheres.

**Table 1:** Crystallographic data and refinement statistics for IgG1 Fc^GlcNAc^

	Fc^GlcNAc^
*Data collection*	
Beamline	Diamond I03
Resolution (Å)	50.0–2.49 (2.59–2.49)^a^
Space group	*C*2
Cell dimensions and angles	
*a*, *b*, *c* (Å)	69.3, 110.9, 77.5
β, α, γ (°)	107.9, 90, 90
Wavelength (Å)	0.976
Unique reflections	19,399 (1923)
Completeness (%)	99.9 (99.9)
*R*_merge_ (%)^b^	0.108 (0.666)
*I*/σ*I*	19.5 (2.2)
Average redundancy	8.4 (3.7)

*Refinement*	
Resolution range (Å)	34.3–2.49 (2.56–2.49)
Number of reflections	18,408 (1348)
*R*_work_ (%)^c^	19.5
*R*_free_ (%)^d^	23.4
r.m.s.d. bonds (Å)	0.008
r.m.s.d. angles (°)	1.2
Atoms per asymmetric unit (protein/water/glycan)	3266/25/14
Average *B*-factors (protein/water/glycan) (Å^2^)	77.3/60.9/104.0
Ramachandran plot^e^ (favored/allowed) (%)	97.7/2.3

a Numbers in parentheses refer to the relevant outer-resolution shell.

b *R*_merge_ = ∑*_hkl_*∑*_i_*|*I*(*hkl*;*i*) − 〈*I*(*hkl*)〉|/∑*_hkl_*∑*_i_I*(*hkl*;*i*), where *I*(*hkl*;*i*) is the intensity of an individual measurement and 〈*I*(*hkl*)〉 is the average intensity from multiple observations.

c *R*_work_ = ∑*_hkl_*||*F*_obs_| − *k*|*F*_calc_||/∑*_hkl_*|*F*_obs_|.

d *R*_free_ is calculated as for *R*_work_, but using only 5% of the data that were removed prior to refinement.

e Ramachandran plots were calculated with MolProbity.^28^
