# Supplementary material for: Selective Deactivation of Serum IgG: A General Strategy for the Enhancement of Monoclonal Antibody Receptor Interactions
Source: J Mol Biol. 2012 Jun 29;420(1-2):1–7. doi: 10.1016/j.jmb.2012.04.002 (PMC3437440; doi:10.1016/j.jmb.2012.04.002)
Supplement: Supplementary file 1 — Supplementary materials [file mmc1.docx]

Supplementary Information for:

Selective Deactivation of Serum IgG: A General Strategy for the Enhancement of Monoclonal Antibody Receptor Interactions

Kavitha Baruah^1, †^, Thomas A. Bowden^2, †^, Benjamin A. Krishna^1^, Raymond A. Dwek^1^,

Max Crispin^1,*^, Christopher N. Scanlan^1,*^

**METHODS**

**Protein Expression and Purification.** Human IgG1 Fc (residues 240-440, following the mumbering of Edelman *et al.*[^1^](#_ENREF_1); GenBank accession no. J00228) was cloned into the pHLsec vector and transiently expressed in Human Embryonic Kidney cells as previously described[^2^](#_ENREF_2) with DNA mixed with polyethyleneimine (PEI) in a mass ratio of 1:1.5, respectively.

For the crystallographic studies of the endoglycosidase-treated IgG Fc (Fig. 1b), the Man_9_GlcNAc_2_ glycoform was initially obtained by transient expression in Human Embryonic Kidney 293T cells in the presence of 20 μM kifunensine, a class I α-mannosidase inhibitor[^3^](#_ENREF_3)^;^ [^4^](#_ENREF_4), to generate IgG-Fc bearing oligomannose N-linked glycan, Man_9_GlcNAc_2_. Cell supernatant was clarified five days following transfection and IgG-Fc was purified by immobilized metal affinity chromatography using Chelating Sepharose Fast Flow Ni^2+^-agarose beads (GE Healthcare, Buckinghamshire, UK). The Man_9_GlcNAc_2_ glycoform of IgG-Fc was then deglycosylated at 37 ˚C for 12 h using 75 μg/mL Endo H and then purified by size exclusion chromatography. Protein purity was assessed by SDS-PAGE analysis and typical yields of deglycosylated IgG were 20 mg/L cell culture.

For the endoglycosidase resistance assays of IgG Fc (Fig. 2), the Man_5_GlcNAc_2_ glycoform was obtained by the transiently expression of the IgG Fc pHLSec construct in GlcNAc transferase I-deficient Human Embryonic Kidney 293S cells[^6^](#_ENREF_6).

Full length IgG1 antibodies bearing human IgG1 Fc domains was generated by cloning the Fab domains of the murine monoclonal antibody CIIC1[^5^](#_ENREF_5) (Developmental Studies Hybridoma Bank, University of Iowa, Department of Biology, Iowa City, IA 52242) into pFUSE-CHIg-hG1 vector (Invivogen, San Diego, California, U.S.A.). Intact CIIC1 antibody with oligomannose-type glycans was transiently expressed in GlcNAc transferase I-deficient Human Embryonic Kidney 293S cells[^6^](#_ENREF_6), as described above for HEK 293T cells, which yields an oligomannose-type glycoform (Man_5_GlcNAc_2_; as confirmed by MALDI-MS). Successful expression of full length antibody was confirmed by SDS-PAGE analysis and also by ELISA assay for binding to mouse collagen Type II protein.

**Enzymatic release of N-linked glycans.** Release of N-linked glycans was performed according to the method of Küster *et al.*[^7^](#_ENREF_7). Oligosaccharides were released from bands containing approximately 10 μg of target glycoprotein that were excised from Coomassie blue-stained reducing SDS-PAGE gels[^7^](#_ENREF_7), washed with alternating water and acetonitrile and dried in a vacuum centrifuge, followed by rehydration with 100 Units/ml of PNGase F (New England Biolabs, MA, U.S.A.) and incubation for 12 hours at 37˚C. The enzymatically released *N*-linked glycans were eluted with water. Endoglycosidase digestion of glycans was performed by addition of 1 μg of recombinant Endo S (Purchased from Genovis AB, Lund, Sweden and also obtained from Professor Ben Davis, CRL, University of Oxford) or 1μl of Endo H (500U/µl, New England Biolabs, MA, U.S.A.) and incubation for 12 hours at 37˚C.

**Matrix-assisted laser desorption/ionization (MALDI) time-of-flight (TOF) mass spectrometry.** Aqueous solutions of the glycans were cleaned with a Nafion 117 membrane[^8^](#_ENREF_8). Positive ion MALDI-TOF mass spectra were recorded with a Shimadzu AXIMA TOF^2^ MALDI TOF/TOF fitted with delayed extraction and a nitrogen laser (337 nm). The acceleration voltage was 20 kV; the pulse voltage was 3200 V; and the delay for the delayed extraction ion source was 500 ns. Samples were prepared by adding 0.5 μL of an aqueous solution of the sample to the matrix solution (0.3 μL of a saturated solution of 2,5-dihydroxybenzoic acid in acetonitrile) on the stainless steel target plate and allowing it to dry at room temperature. The sample/matrix mixture was then re-crystallized from ethanol.

**FcγRIIIa binding assays.** FcγRIIIa (158Val variant; R&D systems, Minneapolis, U.S.A.) at 2.5 μg/mL in PBS was coated on high-binding mircotitre plates (3690, Corning, NY, U.S.A.) overnight at 4˚C. Coated plates were washed with PBS containing 0.05% Tween 20 (Sigma-aldrich, U.S.A.) and blocked for 2 hours at room temperature with 3% BSA in PBS. Serial dilutions of human serum (H4522, Sigma-Aldrich, U.S.A.) or recombinant human IgG1 glycoforms bearing Man_9_GlcNAc_2_ or Man_5_GlcNAc_2_ (starting concentration of 0.1 mg/mL in PBS), was then added and allowed to bind for 2 hours at room temperature. Plates were washed five times with PBS containing 0.05% Tween and binding was detected using a HRP conjugated Fab fragment specific for murine IgG Fab (ab98659, Abcam, Cambridge, UK). TMB substrate (Thermo Scientific, Rockford, IL, U.S.A.) was used for colour development according to manufacturer’s directions. Colour development was stopped by the addition of 2M H_2_SO_4_ and absorbance was measured at 450 nm on a Spectramax M5 (Molecular Devices, California, U.S.A.) multiwall plate reader. For **Figure 1c**, serum was incubated overnight with 1µg/mL of Endo S or PBS at 37˚C. For **Figure 3a**, 1:5 dilution of serum was incubated with 1:100 dilution of Endo S (1 mg/mL) or Endo H (500 U/µL) overnight at 37˚C. Control serum samples were mock treated with PBS and incubated overnight at 37˚C. Data was processed and plotted using Prism (GraphPad software, California, U.S.A.). Apparent affinity was calculated as the concentration of oligomannose mAb corresponding to half-maximal binding on the ELISA binding curve.

**Molecular graphics**

All molecular representations were produced with Pymol (<http://www.pymol.org>).

**Supplementary References**

1. Edelman, G. M., Cunningham, B. A., Gall, W. E., Gottlieb, P. D., Rutishauser, U. & Waxdal, M. J. (1969). The covalent structure of an entire γG immunoglobulin molecule. *Proceedings of the National Academy of Sciences of the United States of America* **63**, 78-85.

2. Aricescu, A. R., Lu, W. & Jones, E. Y. (2006). A time- and cost-efficient system for high-level protein production in mammalian cells. *Acta Crystallogr. D Biol. Crystallogr.* **62**, 1243-1250.

3. Chang, V. T., Crispin, M., Aricescu, A. R., Harvey, D. J., Nettleship, J. E., Fennelly, J. A., Yu, C., Boles, K. S., Evans, E. J., Stuart, D. I., Dwek, R. A., Jones, E. Y., Owens, R. J. & Davis, S. J. (2007). Glycoprotein structural genomics: solving the glycosylation problem. *Structure* **15**, 267-73.

4. Elbein, A. D., Tropea, J. E., Mitchell, M. & Kaushal, G. P. (1990). Kifunensine, a potent inhibitor of the glycoprotein processing mannosidase I. *J Biol Chem* **265**, 15599-605.

5. Nandakumar, K. S., Andrén, M., Martinsson, P., Bajtner, E., Hellström, S., Holmdahl, R. & Kleinau, S. (2003). Induction of arthritis by single monoclonal IgG anti-collagen type II antibodies and enhancement of arthritis in mice lacking inhibitory FcγRIIB. *European Journal of Immunology* **33**, 2269-2277.

6. Reeves, P. J., Callewaert, N., Contreras, R. & Khorana, H. G. (2002). Structure and function in rhodopsin: high-level expression of rhodopsin with restricted and homogeneous N-glycosylation by a tetracycline-inducible N-acetylglucosaminyltransferase I-negative HEK293S stable mammalian cell line. *Proc Natl Acad Sci U S A* **99**, 13419-24.

7. Küster, B., Wheeler, S. F., Hunter, A. P., Dwek, R. A. & Harvey, D. J. (1997). Sequencing of *N*-linked oligosaccharides directly from protein gels: in-gel deglycosylation followed by matrix-assisted laser desorption/ionization mass spectrometry and normal-phase high-performance liquid chromatography. *Anal. Biochem.* **250**, 82-101.

8. Börnsen, K. O., Mohr, M. D. & Widmer, H. M. (1995). Ion exchange and purification of carbohydrates on a Nafion(R) membrane as a new sample pretreatment for matrix-assisted laser desorption-ionization mass spectrometry. *Rapid Commun. Mass Spectrom.* **9**, 1031-1034.

**Supplementary Figures**

**b**

**a**


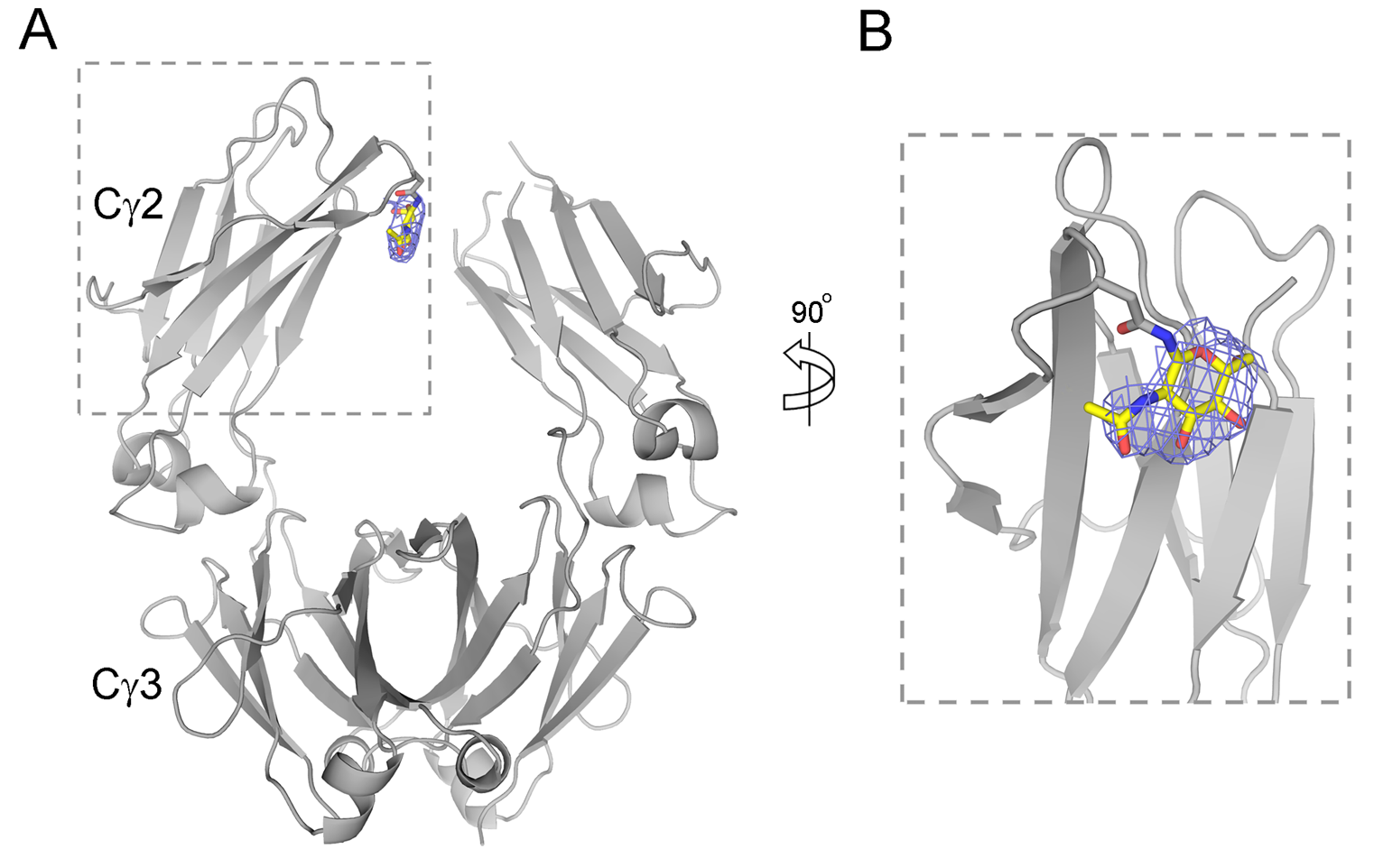


**Figure S1. Crystal Structure of Fc^GlcNAc^**. (**a**) Cartoon representation of homodimeric Fc^GlcNAc^ with GlcNAcβ1−Asn297 structure shown as sticks. The carbons of the GlcNAc are shown in yellow whilst those of side chains are coloured grey. Oxygen atoms are coloured red, and nitrogen atoms are blue. A maximum-likelihood weighted 2*F*_o_-*F*_c_ electron density map is displayed around the GlcNAc residues contoured to 1σ. Note, electron density corresponding to the Asn297-linked GlcNAc and Cγ3-distal loop regions in the Cγ2 domain of the second protomer was not clearly visible and indicates inherent flexibility in these areas. (**b**) Enlarged view of the Cγ2 domain rotated 90 ^o^ around the vertical axis.
